# eEF1A1 promotes colorectal cancer progression and predicts poor prognosis of patients

**DOI:** 10.1002/cam4.4848

**Published:** 2022-05-24

**Authors:** A‐hui Fan, Xiaojuan Zhao, Hao Liu, Danxiu Li, Tongtong Guo, Jiehao Zhang, Lili Duan, Hao Cheng, Yongzhan Nie, Daiming Fan, Xiaodi Zhao, Yuanyuan Lu

**Affiliations:** ^1^ State Key Laboratory of Cancer Biology and National Clinical Research Center for Digestive Diseases, Xijing Hospital of Digestive Diseases Fourth Military Medical University Xi'an China; ^2^ State Key Laboratory of Cancer Biology, Department of Biochemistry and Molecular Biology Fourth Military Medical University Xi'an China; ^3^ Department of Gastroenterology, Tangdu Hospital Fourth Military Medical University Xi'an China; ^4^ Department of Cell Biology, College of Life Science Northwest University Xi'an China

**Keywords:** colorectal cancer, eukaryotic elongation factor 1 A1 (eEF1A1), mitogen‐activated protein kinase (MAPK), proliferation

## Abstract

Colorectal cancer (CRC) is a major leading cause of cancer mortality worldwide in which dysregulated protein synthesis plays an etiologic role. The eukaryotic elongation factor 1 A1 (eEF1A1) exerts significant effects on protein synthesis by contributing to peptide chain extension. Whereas its role in CRC remains to be investigated. In this study, we found that the mRNA and protein levels of eEF1A1 were significantly upregulated in CRC cell lines and tissues. Elevated expression of eEF1A1 was correlated with shorter overall survival in 94 CRC patients. The inhibition of proliferation and cell cycle block were observed in CRC cells after eEF1A1 downregulation. Mechanistically, weighted gene correlation network analysis and further Kyoto Encyclopedia of Genes and Genomes (KEGG) analysis suggested that mitogen‐activated protein kinases (MAPKs) signaling pathways were significantly enriched in high‐eEF1A1 expression group, and the levels of phosphorylated p38/JNK/ERK MAPK were dramatically decreased after eEF1A1 downregulation. Overexpression of eEF1A1 in CRC correlated with a poor prognosis. Collectively, this study determined the oncogenic role of eEF1A1 in CRC proliferation and tumorigenesis. eEF1A1 might be a promising therapeutic target and prognostic biomarker in CRC.

## Graphical abstract

Through combining the analysis of cohorts in GEO database and in vivo and in vitro experiment, we report a novel oncogenic and prognostic role of eukaryotic elongation factor 1 A1 (eEF1A1) in colorectal cancer (CRC).

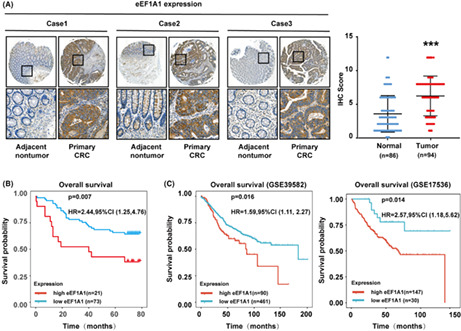

## 1 INTRODUCTION

Colorectal cancer (CRC) is considered as a global health issue with an urgent unmet need of new biomarkers for prognosis prediction and therapeutic intervention.^1^ The prognosis of CRC varies greatly, as the 5‐year survival of patients in early‐stage can exceed 90%, while patients diagnosed with advanced CRC have a dismal prognosis with the 5 year survival of less than 10%.^2^ Therefore, understanding of molecular mechanisms of CRC progression will facilitate the development of innovative effective therapy for cancer treatment.

Increased protein synthesis plays an important role in cancers. Protein synthesis, traditionally involving three essential steps, is restrictedly controlled by a series of regulators.^3^ The eukaryotic elongation factor 1 A1 (eEF1A1), the second‐most abundant protein after actin, is a core subunit of the eukaryotic elongation factor 1 (eEF1) complex.^4^, ^5^, ^6^, ^7^ Besides its canonical function in enzymatic delivery of aminoacyl tRNAs to the ribosome, eEF1A1 was reported to participate in signaling transduction by interacting with key components of the pathway directly and in apoptosis by affecting the synthesis of apoptosis‐related proteins.^4^, ^8^, ^9^, ^10^, ^11^, ^12^ eEF1A1 was reported to promote proliferation by interacting with phospho‐Akt and STAT1 in breast cancer and hepatocellular carcinoma, respectively.^13^, ^14^ Besides, eEF1A1 could enhance migration and invasion in gastric cancer by binding to P21 activated kinase 4.^15^ Although one study suggested that eEF1A1 maybe a positive prognostic factor for CRC, inadequate in vitro and in vivo verifications were performed.^16^ Therefore, it should be of interest to investigate the clinical significance of eEF1A1 and the mechanisms of its function in CRC.

Mitogen‐activated protein kinases (MAPKs) have been implicated in the pathology of cancer and include p38 MAPK, c‐Jun NH2‐terminal kinase (JNK), and extracellular signal‐regulating kinase (ERK).^17^ Cellular stress and proinflammatory cytokines are the main causes of the activation of the p38 and JNK MAPK signaling pathways.^18^, ^19^ The ERK1/2 signaling pathway is activated by growth factors, stimulating cancer cell proliferation and metastasis.^20^, ^21^ The close relationship between MAPK signaling and CRC progression is substantiated by the fact that in about 36% and 9–11% of colorectal cancers, *RAS* mutation and *BRAF* mutation is detected, respectively.^22^ Also, upregulation of the epidermal growth factor (EGF) receptor, which activates downstream ERK/MAPK signaling, frequently occurs in CRC.^2^ Small molecule drugs inhibiting components of MAPK signaling provided a novel strategy for CRC treatment.^23^, ^24^

In our study, increased eEF1A1 expression was determined in CRC cell lines and tissues. The upregulation of eEF1A1 correlated with poor prognosis of CRC patients. The proliferation of CRC cells was significantly reduced by eEF1A1 silencing both in vitro and in vivo. Mechanically, eEF1A1 regulates the activation of MAPK signaling pathways. Collectively, our results demonstrated that eEF1A1 serves as an oncogene and targeting the eEF1A1/MAPK axis may be an effective strategy in CRC.

## 2 MATERIALS AND METHODS

### 2.1 Tissue specimens

HCoIA180Su19, a commercial CRC tissue microarray (TMA) sold by Shanghai Outdo Biotech Company. The TMA contained 86 paired CRC tissues and adjacent normal tissues and eight unpaired CRC tissues. 14 paired CRC tissues and adjacent normal tissues used for detecting eEF1A1 expression were obtained from Xijing Hospital of Digestive Disease. The TMA was collected at 4°C. The freshly resected tissues were collected in liquid nitrogen. Our study received approval from Xijing Hospital's Protection of Human subjects Committee.

### 2.2 Cell culture

All cells used in our study were obtained from the American Type Culture Collection (ATCC), including NCM460, RKO, Caco2, HCT8, SW480, SW620, LOVO. The medium used to culture cells was Dulbecco's modified Eagle's medium (Gibco). The medium was supplementary with 10% bovine growth serum (Gibco BRL). Cells were cultured under an atmosphere with 5% CO_2_ at 37°C.

### 2.3 Western blot analysis

Protein was extracted from cells or tissue as follows: ice‐cold RIPA lysis (Beyotime, China) was supplemented with phosphatase and protease inhibitors and covered cells or tissues on ice for 10 min. The concentration of protein was detected by the BCA Protein Assay Kit (Thermo). SDS‐PAGE was used to separate denatured proteins from cells or tissues. Proteins was transferred to nitrocellulose filter membranes (Millipore). Next, the membrane was incubated with 10% skim milk for 1 h at room temperature and then incubated with the primary and secondary antibodies. The protein band was exposed and detected by a Bio‐Rad Imaging System (Bio‐Rad). The information of antibodies were as follows: anti‐eEF1A1 (1:2000, ab157455; Abcam), anti‐GAPDH (1:2000, #5174; Cell Signaling Technology [CST]), anti‐Cyclin D1 (1:1000, #55506; CST), anti‐Cyclin E1 (1:1000, #4129; CST), anti‐CDK2 (1:1000, #2546; CST), anti‐CDK4 (1:1000, #12790; CST), anti‐Bax (1:1000, ab32503, Abcam), anti‐Bcl‐2 (1:1000, #15071S; CST), anti‐Caspase‐3 (1:800, #9662S; CST), anti‐Cleaved Caspase‐3 (1:800, #9664S; CST), anti‐p38 MAPK (1:2000, #9212S; CST), anti‐p‐p38 MAPK (1:1000, #9216S; CST), anti‐SAPK/JNK (1:1000, #9252S; CST), anti‐p‐SAPK/JNK (1:1000, #9255S; CST), anti‐p44/42 MAPK (ERK1/2) (11,000, #4695S; CST), and anti‐p‐p44/42 MAPK (ERK1/2) (11,000, #4370S; CST). GAPDH was used as the control.

### 2.4 Quantitative Real‐Time PCR

The RNeasy Mini Kit (Qiagen GmbH) was used to extract total RNA. The Prime Script RT Reagent Kit (TaKaRa) was used to reverse transcribed into cDNA. All RT‐qPCR primers were purchased from RiboBio. SYBR Premix ExTaq II (TaKaRa) was used to perform RT‐qPCR assay. An IQ5 multicolor RT‐qPCR detection system was used for quantification. The primer sequences used in this study were summarized in Table 1.

**TABLE 1:** Primer sequences were used in this study

Gene	Forward	Reverse
eEF1A1	5′‐TGTCGTCATTGGACACGTAGA‐3′	5′‐ACGCTCAGCTTTCAGTTTATCC‐3′
CCNE1	5′‐AAGGAGCGGGACACCATGA‐3′	5′‐ACGGTCACGTTTGCCTTCC‐3′
CCND1	5′‐GCTGCGAAGTGGAAACCATC‐3′	5′‐CCTCCTTCTGCACACATTTGAA‐3′
CDK2	5′‐GTACCTCCCCTGGATGAAGAT‐3′	5′‐CGAAATCCGCTTGTTAGGGTC‐3′
CDK4	5′‐TCAGCACAGTTCGTGAGGTG‐3′	5′‐GTCCATCAGCCGGACAACAT‐3′
GAPDH	5′‐GCACCGTCAAGGCTGAGAAC‐3′	5′‐TGGTGAAGACGCCAGTGG‐3′

### 2.5 Transfection and infection

Small interfering RNAs targeting eEF1A1 (sieEF1A1) were purchased from GenePharma. The sequences of siRNAs transfected into RKO and Caco2 cells were as follows: eEF1A1 sieEF1A1–1 5′‐GCAAGUACUAUGUGACUAU TT‐3′, sieEF1A1–2 5′‐CCGGUAUGGUGGUCACCUU TT‐3′, the corresponding negative control siRNA 5′‐UUCUCCGAACGUGUCACGUTT‐3′. DharmaFECT transfection reagent (Thermo Fisher Scientific) was used to transfect the siRNAs. Perform the operation following the manufacturer's instructions. The 2 siRNA hairpin sequences were used to synthesize lentiviral vectors (LV‐sheEF1A1). After 48 infections, puromycin (MP Biomedicals, CA, USA) was added to screen CRC cells successfully infected for about 2 weeks.

### 2.6 Colony formation assay

1 × 10^3^ successfully transfected RKO and Caco2 cells were seeded in a six‐well plate in triplicate. Cultures were followed for 15 days in complete medium until colonies became visible. After incubation with anhydrous ethanol for 5 min, visible colonies were stained with 0.5% crystal violet for another 5 min. GelCOUNT (Oxford Optronix) was used to photograph and count the colonies.

### 2.7 Cell counting kit‐8 assay

Cell counting kit‐8 (CCK‐8) was purchased from Dojindo in order to determine the viability of CRC cells. Cells were seeded at a density of 1 × 10^3^ per well and cultured in a 96‐well plate. At 0, 1, 2, 3, 4, 5 day, we incubated cells with a CCK‐8 solution for another 120 min. The absorbance of each well was detected at 450 nm by VARIOSKAN FLASH (Thermo Fisher Scientific).

### 2.8 Immunohistochemistry

The sides were heated on a 65°C heating panel for 60 min, then deparaffinized, incubated with 3% hydrogen peroxide, blocked overnight at 4°C by avidin‐biotin. After incubation with the following primary antibodies: rabbit anti‐eEF1A1 antibody (1:800, ab157455; Abcam), and mouse anti‐human Ki‐67 (1:1000, #9449S; CST), the sides were then incubated with horseradish peroxidase‐conjugated secondary antibodies (ZSGB). The staining percentage was divided into four grades: <0, 5%; 1, 6–24%; 2, 25%–49%; 3, 50%–74%; 4, 75–100%. The intensity of staining was divided as follows: 0, negative; 1, weak; 2, moderate; 3, strong. The staining intensity score multiplied by the percentage score is equal to the final histological score. The cut‐off value for defining high and low eEF1A1 expression was chosen as 8.

### 2.9 Flow cytometry

Trypsin was used to harvest successfully transfected cells. After fixation with 75% cold ethanol overnight, cells were incubated with RNase water for 30 min. Next, propidium iodide was used to stain cells on ice and in dark for more 30 min. MODFIT software (BD) was used to quantify cell cycle distribution.

### 2.10 In vivo tumorigenicity assay

Briefly, 2 × 10^6^ CRC cells successfully infected with LV‐sheEF1A1–1, LV‐sheEF1A1–2, or negative control were implanted into the flanks of 6‐week‐old nude mice (supplied by the Experimental Animal Center of the Fourth Military Medical University). The tumor volumes were measured three times a week. Mice were sacrificed at 4 weeks. The subcutaneous tumors removed were used to perform histological analysis. The Fourth Military Medical University Animal Care Committee approved all protocols for animal studies.

### 2.11 Microarray and RNA‐sequencing datasets

We obtained mRNA expression profiles of CRC from GSE18105, GES21510, and GSE44076 in the Gene Expression Omnibus (GEO) database. GES21510 and GSE44076 have CRC samples including carcinoma and adjacent tissue, and GSE18105 has CRC paired samples. The Robust Multiarray Average (RMA) method in the *Affy* R package was used to normalize the data.^25^ RNA‐sequencing data of CRC were downloaded from The Cancer Genome Atlas (TCGA) website (https://portal.gdc.cancer.gov/repository). Edger R package was used to normalize the RNA‐sequencing data; log2 transformations were performed for all expression data.

### 2.12 Weighted Gene Coexpression Network analysis

To uncover the significantly correlated gene modules between high and low expression groups in patients with CRC, Co‐expression network analyses were performed as previously described.^26^ Firstly, the pickSoft Threshold function was used to determine the soft thresholding power. The adjacency function was used to create a matrix of adjacencies. Through Person correlation values, the concordances of expression between gene pairs were determined. Next, utilizing the Topological Overlap Matrix (TOM) function, we clustered TOM matrix of all genes. The cutreeDynamic function was used to generate a cluster dendrogram. Finally, we calculated the correlation between clinical characteristics and modules and select the module significantly correlating with high‐expression of eEF1A1 for further study.

### 2.13 Statistical analysis

All data were analyzed using SPSS 22.0. Data were presented as the means ± standard deviation (SD). Student's *t* test (two‐tailed), Pearson correlation coefficients, ANOVA (Dunnett's or LSD post hoc test) or *χ* ^2^‐tests were used to assess statistical significance. For survival analysis, the best cut‐off values for the subgroups were generated by X‐tile software and the statistical significance of difference was determined by the Log‐rank test. Significant differences were considered when *p < 0.05, **p < 0.01, ***p < 0.001.

## 3 RESULTS

### 3.1 Increased eEF1A1 expression in CRC cell lines and tissues

**FIGURE 1:**
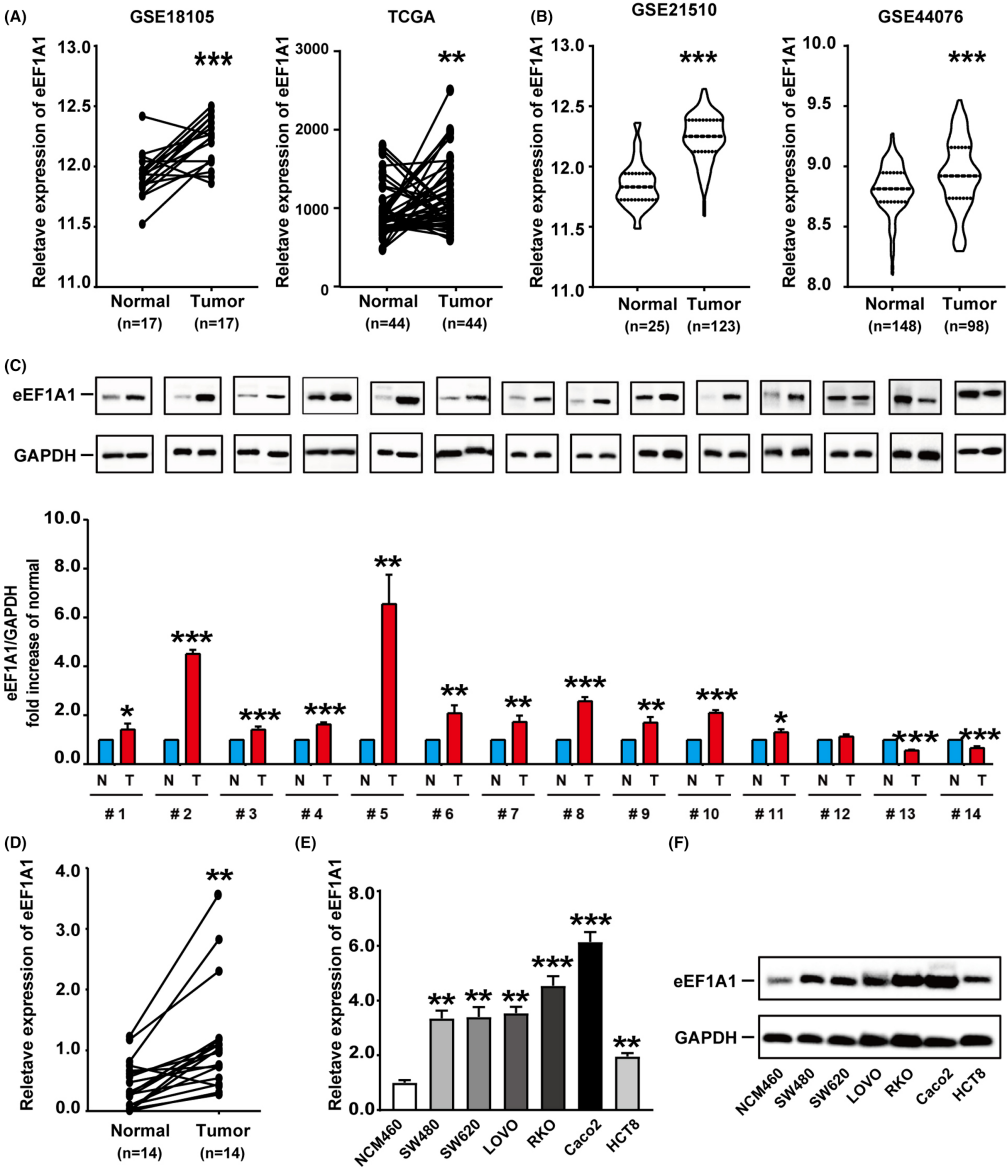
Increased eEF1A1 expression in colorectal cancer (CRC) cell lines and tissues. (A) eEF1A1 expression in paired CRC samples from GSE18105 and TCGA database. (B) eEF1A1 expression in CRC samples from GES21510 and GSE44076. (C) The protein levels of eEF1A1 in 14 paired CRC and adjacent non‐tumorous tissues. The fold change accordingly was used to calculate the average eEF1A1 expression levels in CRC and adjacent non‐tumors. The ratio of eEF1A1/GAPDH of the gray values was used to normalize the protein expression of eEF1A1. Quantitative data represent mean ± SD. (D) The mRNA levels of eEF1A1 in 14 pairs of CRC and corresponding adjacent non‐tumor tissues. (E) The mRNA expression levels of eEF1A1 in the NCM460 and CRC cell lines SW480, SW620, LOVO, RKO, Caco2, and HCT8. The assay repeated three times. (F) The protein expression levels of eEF1A1 in NCM460 and CRC cell lines SW480, SW620, LOVO, RKO, Caco2, and HCT8.

To investigate the expression pattern of eEF1A1 in CRC, the mRNA expression profiles from GSE18105 in the Gene Expression Omnibus (GEO) database and The Cancer Genome Atlas (TCGA) database were analyzed. Compared with adjacent normal tissues, the mRNA expression level of eEF1A1 was remarkedly upregulated in paired CRC tissues (Figure 1A). The same result was obtained when comparing CRC tissues and normal tissues in GES21510 and GSE44076 (Figure 1B). Furthermore, eEF1A1 protein and mRNA levels were investigated in 14 fresh CRC tissue and adjacent normal tissues. The data showed that eEF1A1 expression was upregulated in 11 out of 14 patients (Figure 1C,D). In addition, eEF1A1 expression in NCM460 and CRC cell lines were further detected and the result demonstrated eEF1A1 expression in CRC cells was significantly upregulated compared to that in NCM460 at both the protein and mRNA levels (Figure 1E,F). Collectively, these results suggested that eEF1A1 was remarkedly increased in CRC and may play tumor promoting role in CRC.

### 3.2 eEF1A1 promotes CRC cells proliferation in vitro

**FIGURE 2:**
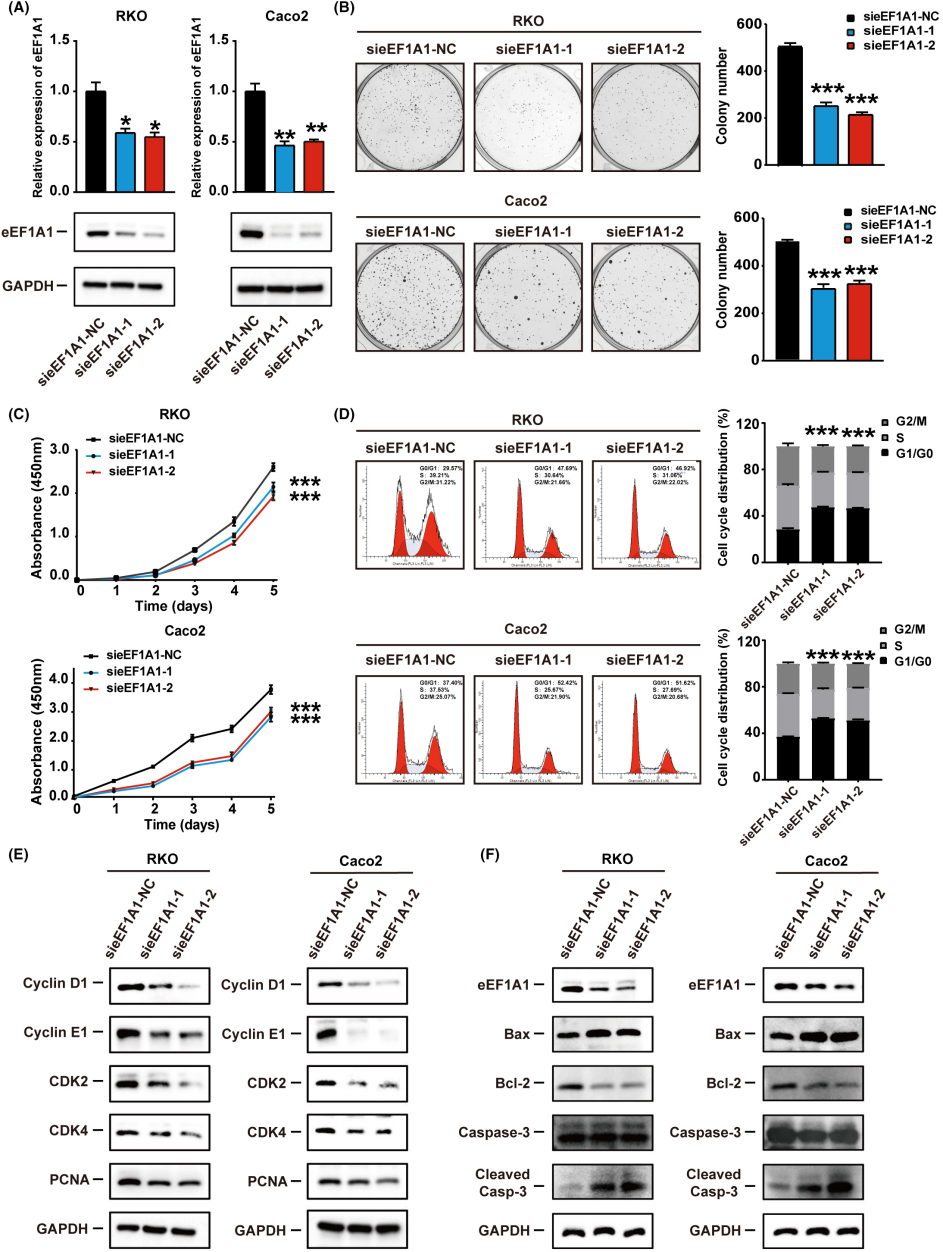
eEF1A1 promotes colorectal cancer cell proliferation in vitro. (A) siRNAs against eEF1A1 (sieEF1A1) were used to knock down eEF1A1 in RKO and Caco2 cells. The silencing of eEF1A1 were confirmed by Western blot and qRT‐PCR. (B) The cell viability of eEF1A1‐knockdown RKO and Caco2 cells was determined by colony formation assays. (C) The proliferation capacity of eEF1A1‐knockdown RKO and Caco2 cells was detected by CCK‐8 assay. (D) The cell cycle distribution of successfully transfected RKO and Caco2 cells. (E) The protein levels of Cyclin D1, Cyclin E1, CDK2, and CDK4 in eEF1A1‐knockdown RKO and Caco2 cells. (F) The protein levels of Bax, Bcl‐2, Caspase‐3, and Cleaved Caspase‐3 in eEF1A1‐knockdown RKO and Caco2 cells.

Based on the eEF1A1 expression levels detected in CRC cell lines, high eEF1A1‐expressing RKO and Caco2 cells were chosen to knock down the eEF1A1 expression by transfecting small interfering RNAs targeting eEF1A1 (sieEF1A1). eEF1A1 silencing was further confirmed by Western blot and RT‐qPCR assays (Figure 2A). To evaluate the effect of eEF1A1 on the proliferation of CRC, in vitro assays were conducted. CCK‐8 assays and colony formation assays indicated that eEF1A1 knockdown significantly inhibited RKO and Caco2 cell proliferation (Figures 2B,C). It has been reported that eEF1A1 depletion could induce the G1/G0 cell cycle arrest.^14^, ^27^ To further investigate the effect of eEF1A1 on the cell cycle, flow cytometry was conducted. The number of cells was significantly increased at G1 phase and markedly decreased at S phase in eEF1A1‐silencing RKO and Caco2 cells (Figure 2D). More importantly, eEF1A1 silencing altered the expression pattern of positive regulators indispensably in cell cycle. Downregulated expression of Cyclin E1, Cyclin D1, CDK2, and CDK4 was detected by Western blot and RT‐qPCR in eEF1A1‐silenced RKO and Caco2 cells (Figures 2E; Figure S1A‐1,B). Knockdown of eEF1A1 also decreased the expression of the proliferation marker PCNA (Figure 2E). Additionally, we detected the expression of apoptosis‐related proteins. Upregulated Cleaved‐Caspase 3 and Bax expression, and downregulated Bcl‐2 expression were detected upon knockdowning of eEF1A1, indicating that eEF1A1 could also repress apoptosis in CRC cells (Figures 2F; Figure S1C). These results demonstrated that the eEF1A1 protein could promote CRC cells proliferation and influence the cell cycle in vitro.

### 3.3 eEF1A1 induces CRC tumorigenesis in vivo

**FIGURE 3:**
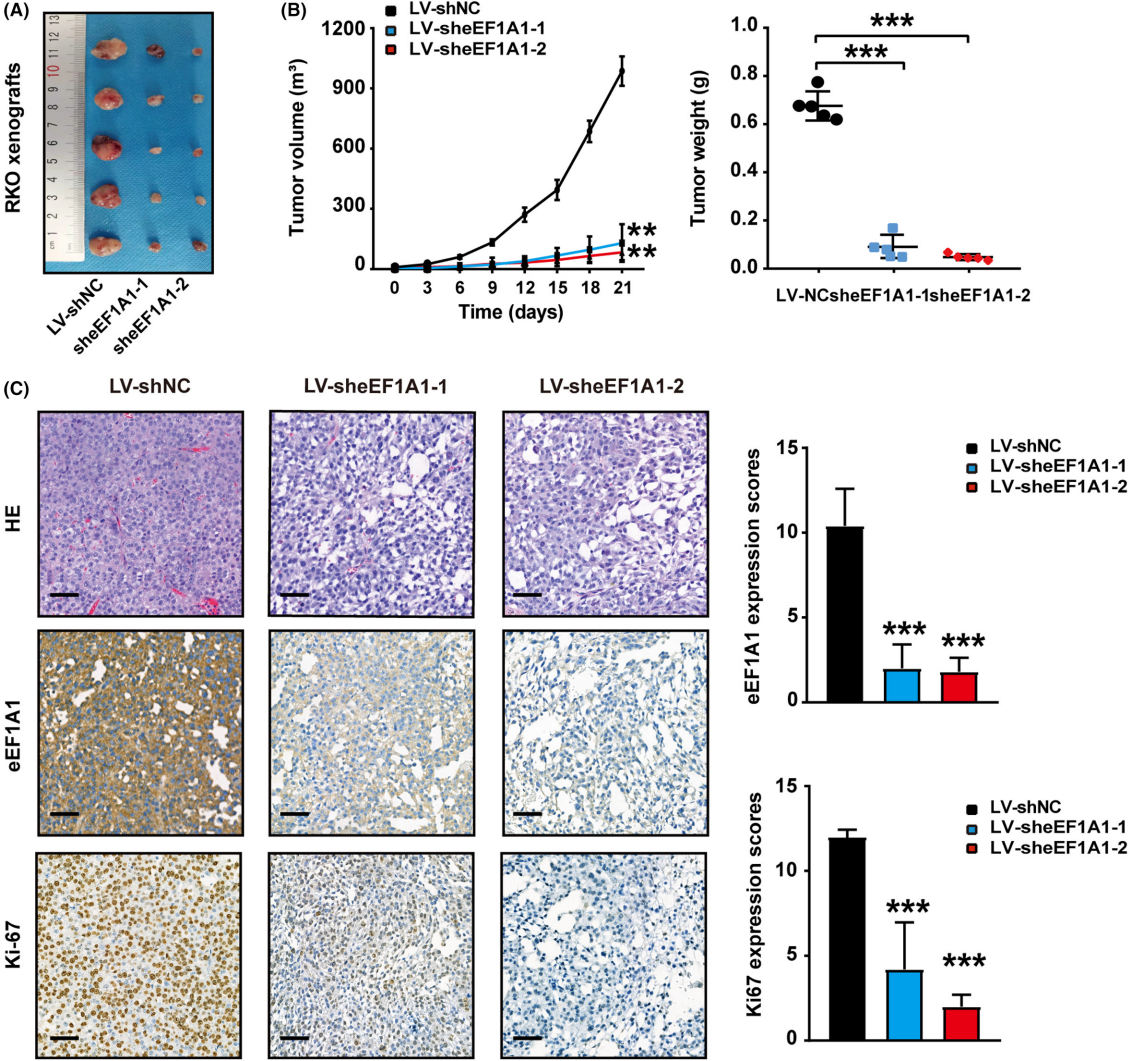
eEF1A1 induces colorectal cancer tumorigenesis in vivo. (A) Photos of xenograft tumors derived by injecting RKO‐sheEF1A1‐1 cells, RKO‐sheEF1A1‐2 or RKO‐sheEF1A1‐NC cells. (B) The xenograft tumors volume was measured every 3 days, tumor growth curves (left) and tumor weight (right) are shown. (C) HE, IHC staining of eEF1A1 and Ki‐67 in the xenograft are shown. Scale bars, 50 μm.

To explore the effect of eEF1A1 on inducing CRC tumorigenesis in vivo, two lentiviruses expressing eEF1A1 shRNAs (LV‐sheEF1A1–1 and LV‐sheEF1A1–2) were used to infect RKO cells. Control cells and RKO‐sheEF1A1 cells were implanted into the flanks of nude mice to generate xenografts (Figure 3A). The tumor volumes were measured once every 3 days. Tumors derived from eEF1A1‐silenced RKO cells grew slower, and the weights were significantly lower than in control mice at the fourth week (Figure 3B). Immunohistochemistry staining of eEF1A1 and Ki‐67 in the xenograft were shown in Figure 3C, and the data supported the tumor promoting role of eEF1A1 in CRC. Together, these results suggested that eEF1A1 could promote CRC tumor growth in vivo.

### 3.4 eEF1A1 actives MAPK signaling in CRC cell lines

**FIGURE 4:**
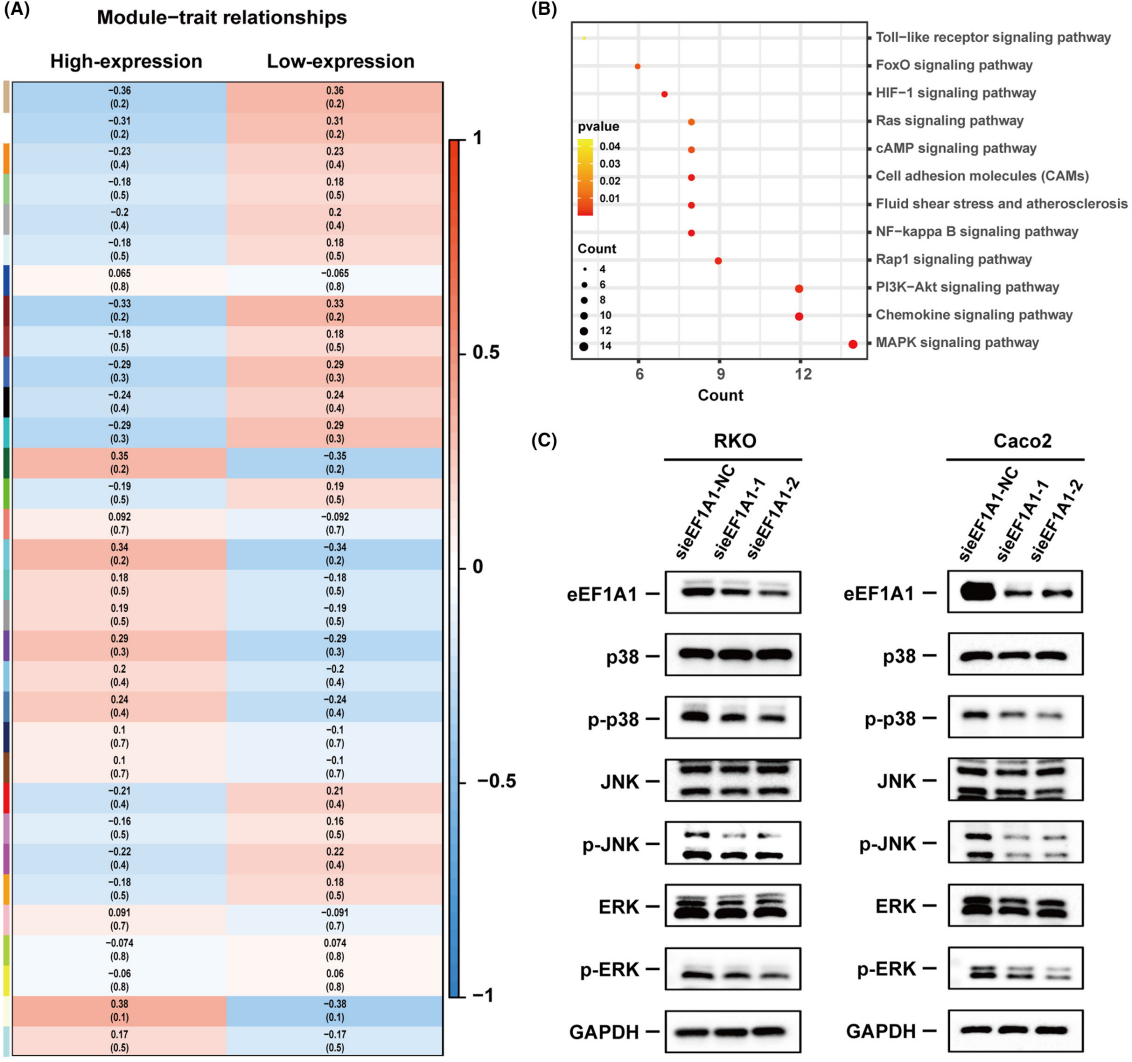
eEF1A1 actives MAPK signaling in colorectal cancer cells. (A) Heatmap of module‐trait relationships. The tan module block is selected for subsequent analysis. (B) KEGG analysis of genes in tan module block. Color depth indicates the enrichment significance, circle size presents the enriched gene count. (C) The protein levels of MAPK signaling pathways related was detected by western blot in eEF1A1‐knockdown RKO and Caco2 cells.

To investigate the underlying mechanisms of eEF1A1 in promoting CRC proliferation, The mRNA expression profiles in GSE18105 were analyzed by weighted gene correlation network analysis (WGCNA) to identify the core genes highly correlated with eEF1A1. The soft power for network construction was set as 5 and the minimum module size was 30 (Figure S2A). We totally excavated 32 modules in total (Figure S2B). To find the module significantly correlated with high‐expression of eEF1A1, the module‐trait correlation coefficients were calculated. The data suggested that the tan color module higher correlated with high‐expression of eEF1A1 (Figure 4A). Next, for further investigating the underlying biological pathways correlating high‐expression of eEF1A1 trait, the top genes of the tan module were analyzed by Kyoto Encyclopedia of Genes and Genomes (KEGG) enrichment analysis. As shown in Figure 4B, MAPK signaling pathway was significantly enriched. The western blot analysis further confirmed that knockdown of eEF1A1 expression decreased the phosphorylation levels of ERK, p38, and JNK MAPK in Caco2 and RKO cells (Figure 4C; Figure S2C). Collectively, eEF1A1 promotes progression of CRC via regulating MAPK signaling pathway.

### 3.5 Overexpression of eEF1A1 in CRC correlated with a poor prognosis

**FIGURE 5:**
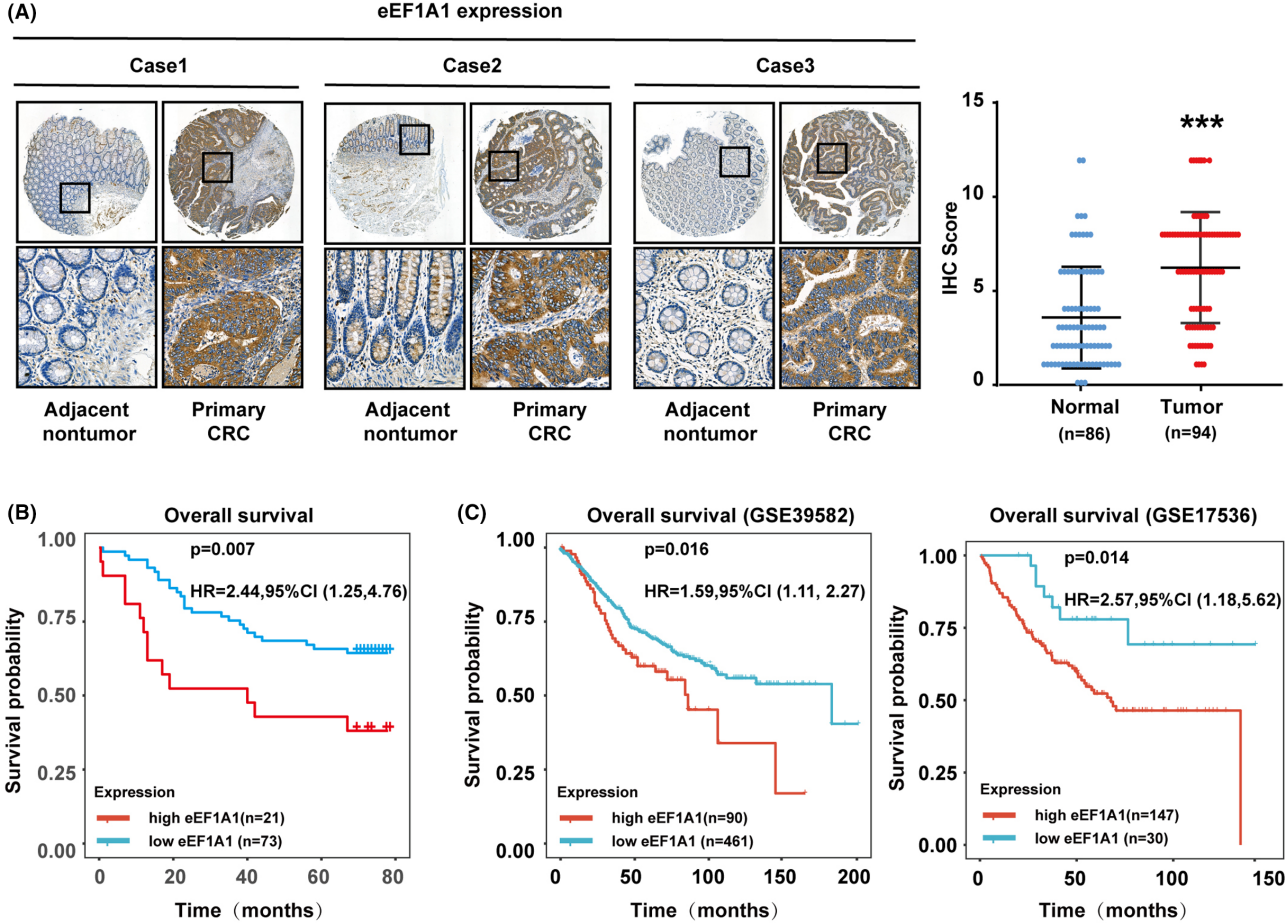
Overexpression of eEF1A1 in colorectal cancer (CRC) correlated with a poor outcome. (A) eEF1A1 expression in paired CRC samples from TMA cohort performed. Scale bars, 600 μm (above) and 40 μm (below). Representative images (left) and IHC score grades of eEF1A1 expression are shown (right). (B) Correlation between eEF1A1 expression and OS in a TMA cohort. (C) Correlation between eEF1A1 expression and OS in GSE39582 and GSE17536.

A tissue microarray (TMA) was used to confirm the expression profile and explore the prognosis implication of eEF1A1 in CRC. eEF1A1, primarily located in the cytoplasm, was remarkably upregulated in CRC tissues compared to normal tissues (Figure 5A). Next, to explore the prognostic implication of eEF1A1 in CRC, the connection between the expression of eEF1A1 and OS was analyzed by Kaplan–Meier analysis. The result suggested that patients exhibiting high eEF1A1 expression had poorer prognosis (Figure 5B). We further determined the connection between eEF1A1 expression and prognosis from GSE17536 and GSE39582 data, the GEO results supported that upregulated eEF1A1 expression correlated with shorter OS in CRC (Figure 5C). In conclusion, overexpression of eEF1A1 in CRC correlated with a poor prognosis.

## 4 DISCUSSION

Given connections between tumor proliferation and the progression of tumor are well‐established, uncovering the underlying mechanism will contribute to the prevention and helps to provide a novel strategy for cancer therapy.^28^ mRNA translation plays a significant role in tumor proliferation. eEF1A1, a highly conserved GTP‐binding protein, promotes protein synthesis by extending peptide chain whereby we postulate that eEF1A1 overexpression may be critical to fuel neoplastic growth.^29^ In our study, eEF1A1 expression proved to be markedly increased in CRC cell lines and tissues, and higher expression of eEF1A1 was correlated with shorter OS. eEF1A1 knockdown inhibited tumorigenesis and CRC cell proliferation and caused G1/S cell cycle block. In vivo experiments confirmed the oncogenic role of eEF1A1 in CRC growth. Mechanically, we found that eEF1A1 could activate MAPK signaling pathway. Overall, our data supported that eEF1A1 may be a potential promising biomarker in CRC.

Notably, tumor inhibitors targeting the EF1A complex have been continually developed in the past years. Plitidepsin is an antitumor agent of marine origin. The agent exerts an anti‐proliferative effect by a high‐affinity interaction with the eEF1A1.^30^ GT75 is another tumor inhibitor specially interfering with eEF1A1 activity. GT75 is a nucleotide aptamer containing a GT repetition. The agent could suppress the malignant phenotypes and enhance the effects of bortezomib and idarubicin in hepatocarcinoma cells.^31^ We hope that patients with CRC could benefit from the therapies targeting eEF1A1 in the future.

The dysregulation of eEF1A1 is a common etiologic agent in oncogenesis and the development of tumor. However, the underlying mechanisms remain not fully uncovered.^32^, ^33^, ^34^ Liu S. et al. suggested that methylation of eEF1A contributed to oncogenesis by promoting translation elongation and protein synthesis.^35^ Remarkably, emerging evidences suggested that eEF1A1 may also promote tumorigenesis independent of its canonical function in translation elongation but as a transcription factor.^13^, ^34^ For instance, Wu A. et al. found that eEF1A1 could directly target the promoter region of murine double minute 2 to regulate the expression and promote lung cancer metastasis.^36^ In this study, the WGCNA algorithm was used to discover the core genes. The method of using the WGCNA algorithm to discover the core genes by screening clinical feature‐related gene co‐expression networks is well‐established.^37^, ^38^ Further KEGG enrichment analysis identified that MAPK signaling pathway was significantly enriched. Since the ERK MAPK pathway is one of the most important for cell proliferation,^22^ we focused on the relationship between eEF1A1 and MAPK pathway in our subsequent research. The Western blot further confirmed that knockdown of eEF1A1 significantly reduced the phosphorylated forms of ERK1/2, JNK, and p38 MAPK, suggesting that eEF1A1 regulated malignancy phenotypes of CRC cells by promoting the activation of critical proteins in MAPK signaling pathways. The connection between the aberrant expression of eEF1A1 and MAPK signaling pathways remains exclusive. Given the mechanisms revealed in the previous study, one explanation might be that eEF1A1 is a p‐p38/JNK/ERK‐interacting protein which modulates the activity of p‐p38/JNK/ERK and regulates the proliferation of CRC cells.^13^ Overall, the interaction between eEF1A1 and MAPK looks forward to further investigation. We have to admit that we did not exclude the possibility that eEF1A1 mediated CRC proliferation through other mechanisms such as forming a complex with hnRNPE1^39^ and FAT10.^28^

The underlying mechanisms of the aberrant overexpression of eEF1A1 in CRC remain unknown. Previous study has shown that eEF1A1 was degraded by the ubiquitin–proteasome system (UPS).^40^ Meanwhile Human HLA‐F adjacent transcript 10 could antagonize its ubiquitination to promote eEF1A1 stabilize and induce cancer cell proliferation.^28^ Besides, a study suggested that BPOZ‐2 directly interacted with eEF1A1 to induce eEF1A1 ubiquitylation and degradation. Based on these data, one explanation might be that eEF1A1 is stabilized by repressing the ubiquitylation and degradation of eEF1A1. Additionally, emerging evidence has demonstrated that long non‐coding RNAs, which is a class of non‐coding transcripts longer than 200 bases in length, exert critical effects on eEF1A1 regulation.^41^ For instance, lncRNA MALAT1 interacted with the promoter regulatory element of eEF1A1 and affected the status of H3K4 methylation in the gene promoter in breast cancer. miRNAs are significant epigenetic regulators which could regulate the levels of cancer‐related genes expression.^42^ Upstream microRNAs of eEF1A1 we predicted included miR‐199a, miR‐516a, miR‐629, miR‐495, and miR‐581. We are in dire need of further studies to determine the potential upstream molecules.

Taken together, our study revealed for the first time that eEF1A1 promoted CRC proliferation and further uncovered that eEF1A1 played a crucial role in CRC via modulating the activity of p‐p38/JNK/ERK pathway. In addition, according to the survival analysis, overexpression of eEF1A1 in CRC was correlated with a poor prognosis. These data strongly suggested that targeting eEF1A1/MAPK axis may be of potential therapeutic value in CRC. However, we have to admit that the exact mechanisms underlying eEF1A1‐mediated activation of MAPK signaling remained to be investigated.

## 5 CONCLUSION

Our study demonstrated that overexpression of eEF1A1 promoted CRC cell growth and proliferation and that its upregulation was correlated with a poor CRC prognosis. Functional experiments and bioinformatics analyses indicated that eEF1A1 promoted CRC progression by activating MAPK signaling pathways. In conclusion, we reported a novel oncogenic and prognostic role of eEF1A1 in CRC and eEF1A1 might be a promising therapeutic target and a potential prognostic biomarker for CRC.

## AUTHOR CONTRIBUTION

Conceptualization, A‐hui Fan, Xiaojuan Zhao and Danxiu Li; Formal analysis, A‐hui Fan, Xiaojuan Zhao and Jiehao Zhang; Funding acquisition, Yuanyuan Lu, Xiaojuan Zhao and Daiming Fan; Investigation, A‐hui Fan, Xiaojuan Zhao, Tongtong Guo, Lili Duan; Resources, Daiming Fan, Yongzhan Nie and Xiaojuan Zhao; Supervision, Daiming Fan, Xiaojuan Zhao and Yuanyuan Lu; Validation, Yuanyuan Lu; Visualization, Xiaojuan Zhao and Yuanyuan Lu; Writing – original draft, A‐hui Fan and Hao Cheng; Writing – review & editing, Xiaojuan Zhao and Yuanyuan Lu; All authors have read and agreed to the published version of the manuscript.

## CONFLICT OF INTEREST

The authors declare no conflict of interest.

## ETHICAL STATEMENT

This study received approval from Xijing Hospital's Protection of Human subjects Committee. The Fourth Military Medical University Animal Care Committee approved all protocol for the animal studies. All subjects gave written informed consent before their inclusion in this study.

## Supporting information


**FIGURE S1** Relative cell cycle and apoptosis‐related proteins expression in eEF1A1‐knockdown RKO and Caco2 cells. (A) Relative cell cycle‐related protein expression in eEF1A1‐knockdown RKO and Caco2 cells. The data were presented as mean ± SD (*n* = 3). (B) The mRNA expression levels of CCNE1, CCND1, CDK2, and CDK4 in eEF1A1‐knockdown RKO and Caco2 cells were examined by qPCR assay. (C) Relative apoptosis‐related protein expression in eEF1A1‐knockdown RKO and Caco2 cells. The data were presented as mean ± SD (*n* = 3)

**FIGURE S2** Determination of soft‐threshold power and identification of modules. (A) The scale‐free fit index for various soft‐thresholding powers (left) and the mean connectivity for various soft‐thresholding powers (right) are displayed. (B) Dendrogram of all expressed genes clustered. The clustering dendrogram of genes (above) and the assigned module colors (below) are shown. (C) Relative protein expression of p38, p‐p38, JNK, p‐JNK, ERK, and p‐ERK in eEF1A1‐knockdown RKO and Caco2 cells. The data were presented as mean ± SD (*n* = 3)

**FIGURE S3‐1** Original images of immunoblots in this study

FIGURE S3–2

## Data Availability

All data generated or analyzed during this study are included in this published article.
