# Supplementary figures and images for: eEF1A1 promotes colorectal cancer progression and predicts poor prognosis of patients
Source: Cancer Med. 2022 May 24;12(1):513–24. doi: 10.1002/cam4.4848 (PMC9844609; doi:10.1002/cam4.4848)

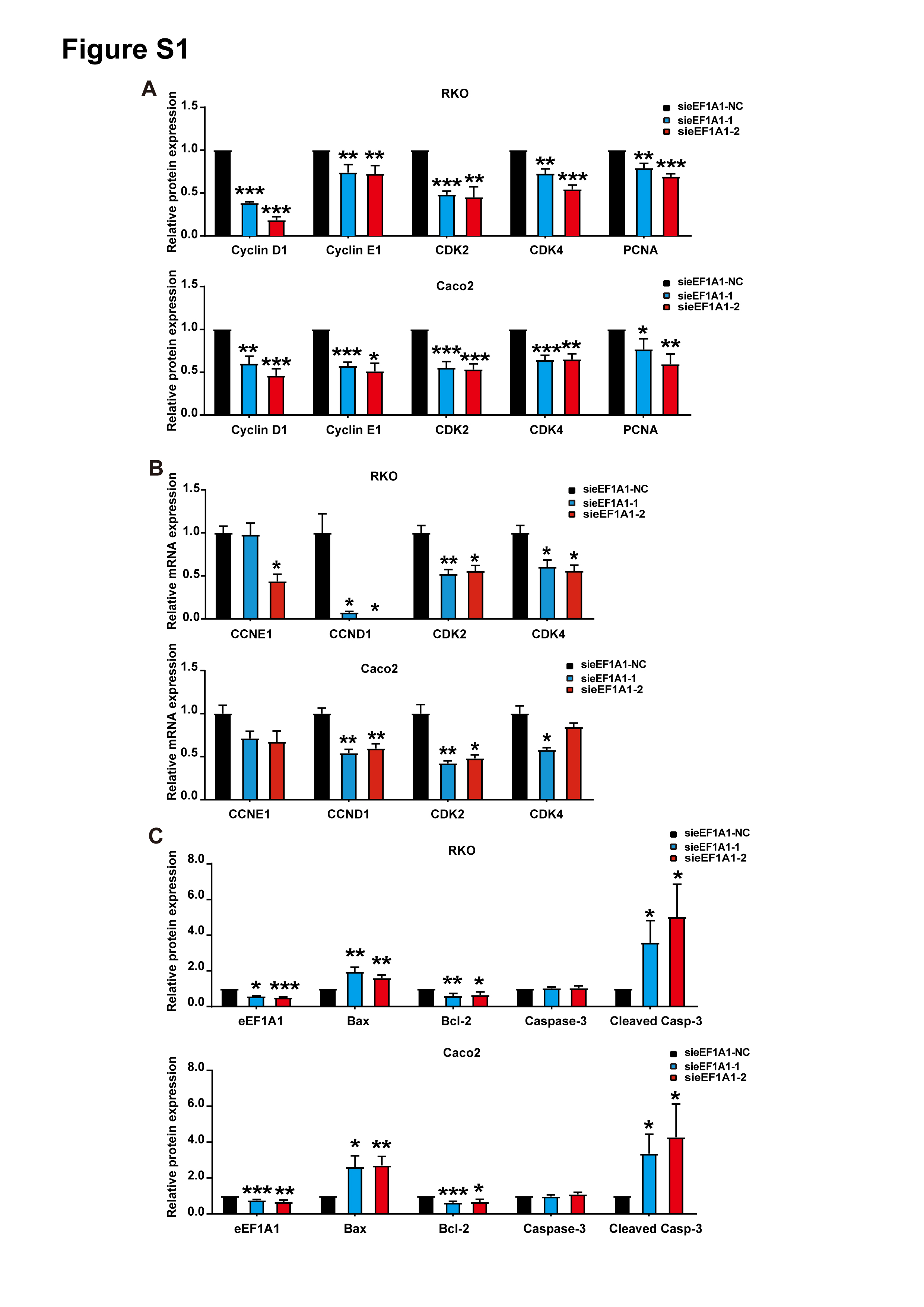

Supplement: Supplementary file 1 — FIGURE S1 Relative cell cycle and apoptosis‐related proteins expression in eEF1A1‐knockdown RKO and Caco2 cells. (A) Relative cell cycle‐related protein expression in eEF1A1‐knockdown RKO and Caco2 cells. The data were presented as mean ± SD (n = 3). (B) The mRNA expression levels of CCNE1, CCND1, CDK2, and CDK4 in eEF1A1‐knockdown RKO and Caco2 cells were examined by qPCR assay. (C) Relative apoptosis‐related protein expression in eEF1A1‐knockdown RKO and Caco2 cells. The data were presented as mean ± SD (n = 3) [file CAM4-12-513-s002.jpg]

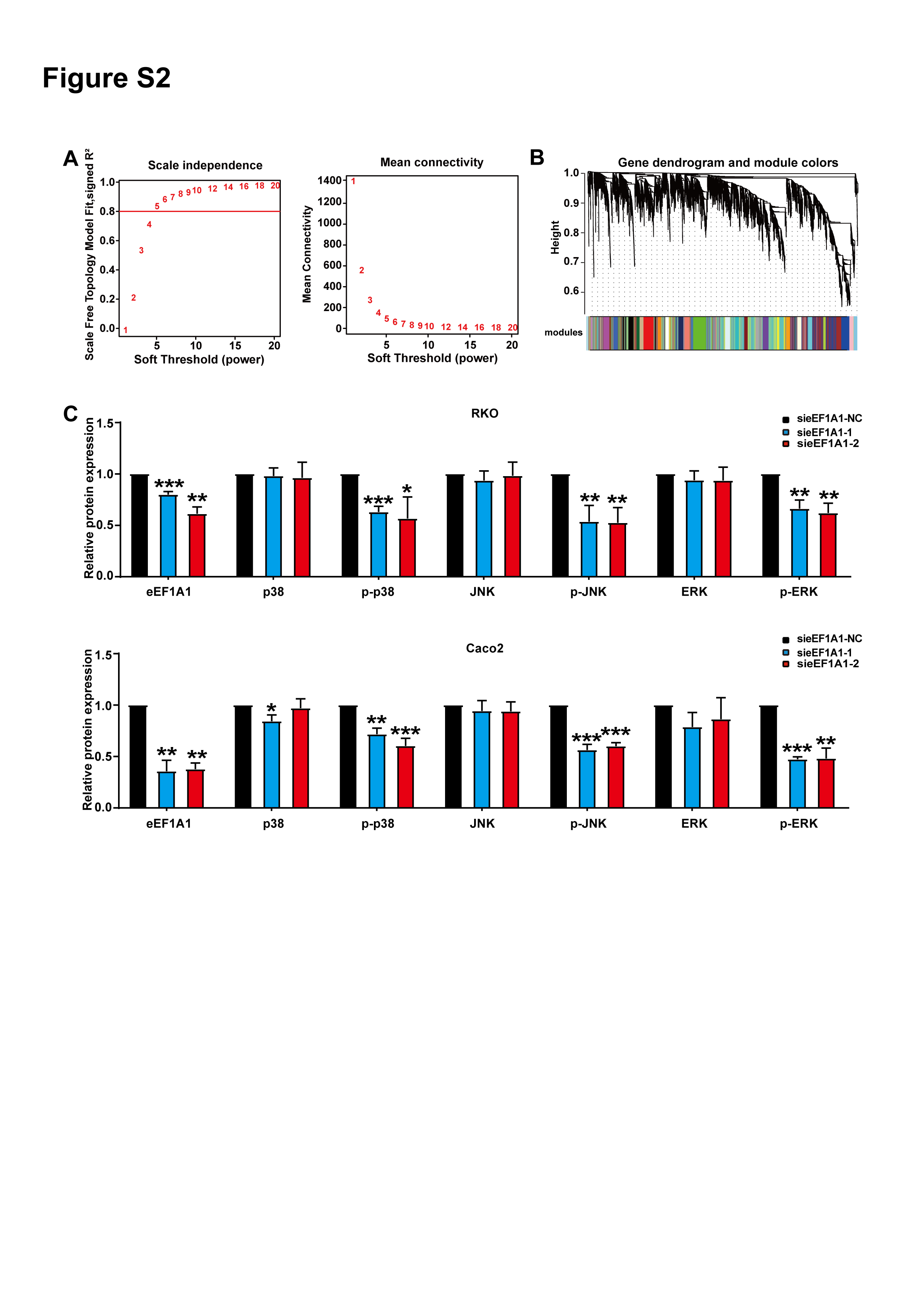

Supplement: Supplementary file 2 — FIGURE S2 Determination of soft‐threshold power and identification of modules. (A) The scale‐free fit index for various soft‐thresholding powers (left) and the mean connectivity for various soft‐thresholding powers (right) are displayed. (B) Dendrogram of all expressed genes clustered. The clustering dendrogram of genes (above) and the assigned module colors (below) are shown. (C) Relative protein expression of p38, p‐p38, JNK, p‐JNK, ERK, and p‐ERK in eEF1A1‐knockdown RKO and Caco2 cells. The data were presented as mean ± SD (n = 3) [file CAM4-12-513-s004.jpg]

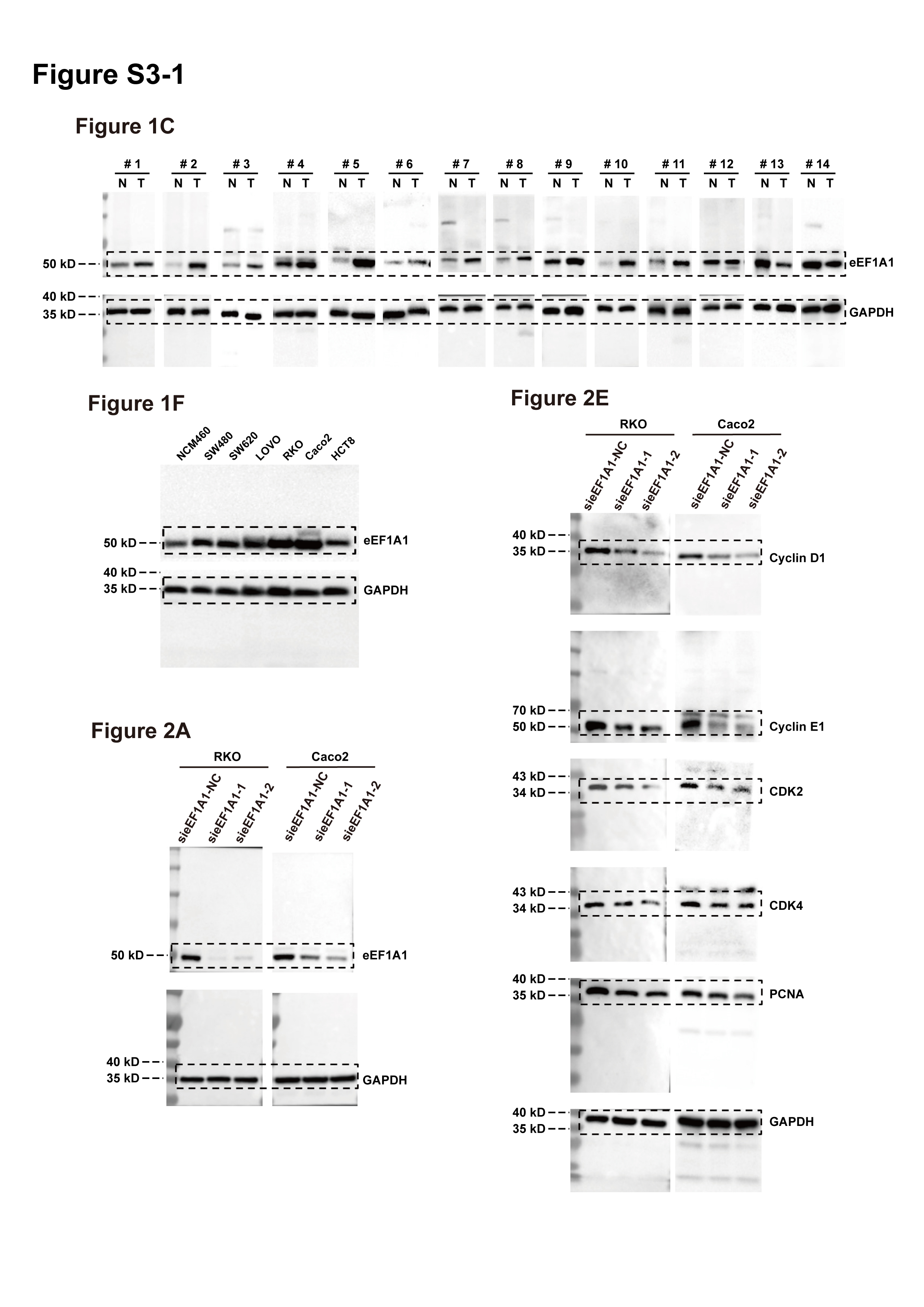

Supplement: Supplementary file 3 — FIGURE S3‐1 Original images of immunoblots in this study [file CAM4-12-513-s001.jpg]

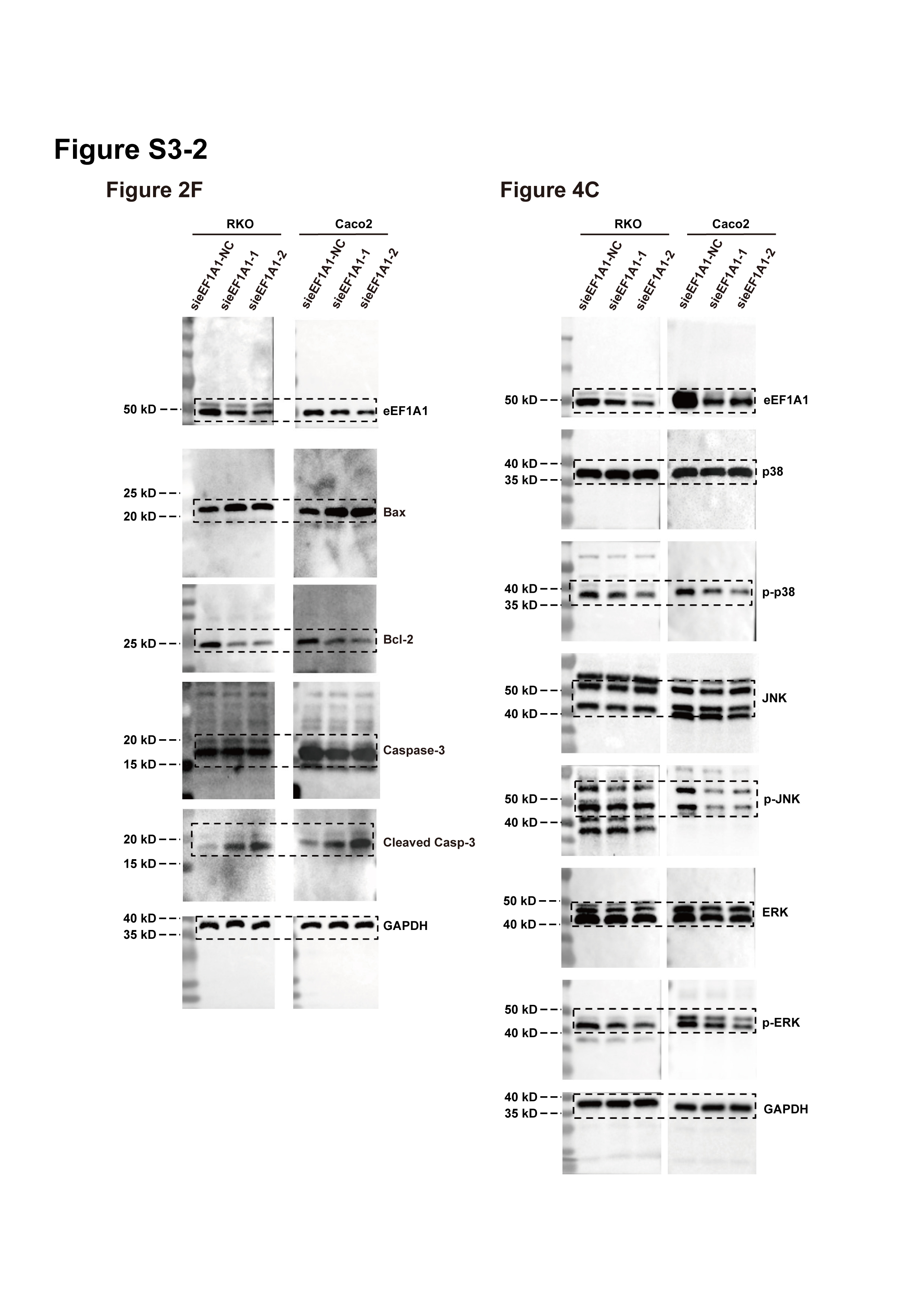

Supplement: Supplementary file 4 — FIGURE S3–2 [file CAM4-12-513-s003.jpg]
